# JCAS Gets Indexed in PubMed

**DOI:** 10.4103/0974-2077.63219

**Published:** 2010

**Authors:** Venkataram Mysore

**Affiliations:** *Venkat Charmalaya - Centre for Advanced Dermatology, Bangalore, Karnataka, India*

I am proud and happy to announce some pleasant news to all readers of Journal of Cutaneous and Aesthetic Surgery (JCAS) and members of ACSI (Association of Cutaneous Surgeons of India). JCAS has been approved for indexing in PubMed-an important milestone in the evolution of any journal. It is a matter of great satisfaction and pride that this has been achieved within two years of starting the journal. This has been accomplished with the cooperation, support and guidance from several sources - the editorial team, both national and international, in particular my deputy editor Dr Nischal KC, all the office-bearers of ACSI (in particular President Dr. Somesh Gupta and former presidents Drs. Niti Khunger, Narendra Patwardhan, and Sharad Mutalik), all the editors and referees; and most importantly all the contributing authors. Our publishers, Dr Sahu and his hardworking staff at Medknow Publications, have been a constant source of support and guidance without whose support this recognition would not have been possible. I express my sincere gratitude to all of them.

JCAS has also started a new initiative - starting with this issue, the journal will carry a FOCUS on a topic of relevance to the cutaneous and aesthetic surgeon.

This issue's FOCUS is on fillers, an important aesthetic modality, with emphasis on different aspects of fillers such as materials of fillers, side-effects, longevity, biofilms and practical tips for the use of fillers. Guest editor Dr Maya Vedamurthy has compiled the invited articles and I wish to thank her for her valuable contribution.

I hope the readers will appreciate this initiative. We welcome any feedback from the authors and readers to enhance the impact factor of the journal. The forthcoming issues in 2010 will include FOCUS on other topics in aesthetic and cutaneous surgery such as acne surgery, hair restoration, vitiligo surgery and teledermatology. I invite authors to contribute articles on these topics.

